# Strengthening healthcare workforce capacity during and post Ebola outbreaks in Liberia: an innovative and effective approach to epidemic preparedness and response

**DOI:** 10.11604/pamj.supp.2019.33.2.17619

**Published:** 2019-05-31

**Authors:** Philip Bemah, April Baller, Catherine Cooper, Moses Massaquoi, Laura Skrip, Julius Monday Rude, Anthony Twyman, Phiona Moses, Redda Seifeldin, Kanagasabai Udhayashankar, Kayla Enrique, Michelle Niescierenko, Chantelle Owen, Lauren Brown, Bonkoungou Boukaré, Desmond Williams, Tolbert Nyenswah, Francis Kateh, Bernice Dahn, Alex Gasasira, Ibrahima Socé Fall

**Affiliations:** 1 National Public Health Institute of Liberia; 2 World Health Organization, Monrovia, Liberia; 3 Ministry of Health, Monrovia, Liberia; 4 Centers for Disease Control and Prevention, Monrovia, Liberia; 5 Academic Consortium Combating Ebola in Liberia, Monrovia,Liberia; 6 International Medical Corps, Monrovia, Liberia; 7 World Health Organization Regional Office for Africa, Brazzaville, Congo

**Keywords:** Healthcare workforce capacity, Ebola outbreak, epidemic preparedness response

## Abstract

**Introduction:**

The 2014-2016 Ebola virus disease (EVD) outbreak in Liberia highlighted the importance of robust preparedness measures for a well-coordinated response; the initially delayed response contributed to the steep incidence of cases, infections among health care workers, and a collapse of the health care system. To strengthen local capacity and combat disease transmission, various healthcare worker (HCW) trainings, including the Ebola treatment unit (ETU) training, safe & quality services (SQS) training and rapid response team (RRT), were developed and implemented between 2014 and 2017.

**Methods:**

Data from the ETU, SQS and RRT trainings were analyzed to determine knowledge and confidence gained.

**Results:**

The ETU, SQS and RRT training were completed by a total of 21,248 participants. There were improvements in knowledge and confidence, an associated reduction in HCWs infection and reduced response time to subsequent public health events.

**Conclusion:**

No infections were reported by healthcare workers in Liberia since the completion of these training programs. HCW training programmes initiated during and post disease outbreak can boost public trust in the health system while providing an entry point for establishing an Epidemic Preparedness and Response (EPR) framework in resource-limited settings.

## Introduction

The 2014-15 Ebola outbreak in West Africa had a devastating effect on the healthcare system, in particular for healthcare workers (HCWs). HCWs were up to 32 times more likely to be infected with Ebola than the general population and two-thirds of infected HCWs died [1, 2] .Multiple factors contributing to EVD transmission among HCWs included deficiencies in administrative, engineering and environmental regulations, overcrowding of public hospitals and unsafe employment conditions. A major driver was the limited infection prevention and control (IPC) capacity in West Africa at the time of the outbreak [1]. Thus, under the incident management system (IMS) of the ministry of health and social welfare (MOHSW) [3], the Case Management technical pillar and the National IPC Task Force took the lead to address these IPC capacity gaps through training [4]. This was done in collaboration with international partners and local non-governmental organizations (NGOs). The training of HCWs in IPC was initiated at the peak of the outbreak as a means to enhance in-country epidemic preparedness and response (EPR). Ultimately, there were two large-scale training initiatives implemented for HCWs during the 2014-2015 Ebola outbreak. These included the keep safe, keep serving (KSKS) and the healthcare workers in Ebola treatment unit program (ETU). The IPC-focused KSKS curriculum [5] was developed for non-ETU settings and was implemented with over 4000 HCWs [6].

Once Liberia was declared free of human-to-human Ebola transmission in May 2015, the government’s focus shifted to sustaining a “resilient zero”. This characterized the third phase of the Ebola response [7] emphasizing health system recovery and strengthening, as reflected in the development of Liberia’s investment plan for building a resilient health system (2015-2021) [8]. The plan aimed to ensure universal health coverage, guarantee health security, and improve health outcomes, while complementing the national health policy and plan (NHPP) (2011-2021). Enhancing safe and high-quality health services through the adherence of HCWs to appropriate clinical care practices was identified as a main investment area. HCWs trained during KSKS on the “No touch policy” had to be reoriented to interact with their patients using standard care procedures, enhanced with reasonable IPC measures- that is, they required an understanding of the importance of implementing IPC measures at all times (known as standard precautions), not just during outbreak settings. Moreover, the EVD crisis had resulted in low confidence in the health system among HCWs and the general public. To address these priorities and issues, the build back better health system strategy (BBBHSS) was developed [9]. This included a training component which became known as safe & quality service (SQS).

By 2016, public confidence in their health care system had been re-established [10]. The MOH and the then newly launched national public health institute of Liberia (NPHIL) undertook the transition from rehabilitation and recovery mode to strengthening epidemic preparedness. To improve, standardize and decentralize preparedness capacity, rapid response teams (RRT) were formed in all 15 counties. Additionally, a national RRT was established to provide support to counties during large public health events. RRT training packages were developed, rolled out and then tested through simulations in each county. The WHO disaster risk management cycle which informs public health emergencies preparedness describes the different phases that Liberia experienced with the EVD outbreak [11]. Typically, the pre-disaster (and required trainings) precedes the post-disaster phases; in Liberia, the cycle was reversed. Here we describe the processes for developing and implementing the HCWs in Ebola treatment unit program during the EVD outbreak response, the SQS training during the post-EVD recovery period and the RRT training undertaken in post-EVD preparedness phase in preparation for future outbreaks. We also present results from pre-and post-tests administered to HCWs who participated in any of the three trainings as a measure of their impact on knowledge and attitudes. These trainings engaged over 21,000 national and international healthcare workers in capacity building to enhance the use of standard and transmission-based prevention measures.

## Methods

**Overview of the healthcare workers in ebola treatment unit:** the objectives of the training on healthcare workers in Ebola treatment unit involved ensuring HCW safety, improving effectiveness and quality of care for patients in the Ebola treatment units (ETUs), and fighting the epidemic through patient care and isolation to prevent further spread. The package, an adoption and adaptation of existing WHO Ebola response training packages [12], included IPC (standard and additional precautions), EVD case management, safe burials, amongst other key components required to work in an ETU (Table 1). The 8 days skills-oriented training course was divided into a clinical and non-clinical stream in a 3-phase format: classroom-based didactic training (phase I), simulated patients (recovered Ebola patients) in a mock ETU (phase 2), and mentored introduction seeing patients in an actual ETU (phase 3). On completion and passing the post training exam participants were awarded certificates [13]. The MOH/IMS mandated the training as a prerequisite for any national or international Ebola outbreak responder planning to work in an ETU. As the training workforce and needs increased, mobile teams were formed by various partners to support and build county health workforce capacity in all the 15 counties. Participants were administered nine-item pre- and post-test questionnaires to assess change in confidence and knowledge upon exposure to the training.

**Table 1:** Healthcare workers in Ebola treatment unit training content

Category	Phase 1 and 2	Phase 3
**General modules**	Overview of Ebola Strategies to stop EVD transmission Infection Prevention and Control PPE for Ebola HCW preparedness for work and Heat stress management	Introduction to ETU (layout, wards, and patient flow) ETU Standard Operating Procedures PPE Demonstration Admissions and discharge protocol
**Clinical stream**	Screening and overall ETU organization Clinical care for Ebola patients Collection, packaging, and transportation of EVD samples	Ward introduction Triage Case management (assessment of fluid and antibiotic needs, IV line placements, medication protocols, prescriptions, and abbreviations) Liberia Ebola clinical guidelines Lab testing
**Non-Clinician stream**	Principles of Cleaning and Disinfection, Chlorine preparation Environmental Cleaning Waste Management Dead body Management	Cleaning and disinfection in the low and high-risk zones Spraying Laundry management Dead body management Incineration

**Figure 1:**
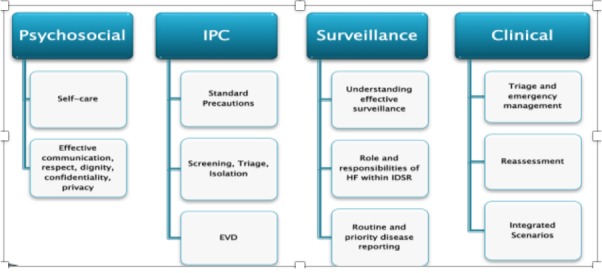
Safe & quality services (SQS) training package content

**Overview of the SQS training package:** the SQS training package, an MoH and WHO initiative, provided an integrated approach to restoring and building healthcare workers confidence, knowledge and skills for delivery of safe and quality health services. The core components of this training package included psychosocial support (PSS), Infection Prevention & Control (IPC), EVD management, surveillance and clinical emergency management (Figure 1). Initially, technical working groups were formed per technical area to review, adopt, and adapt existing training materials (e.g. integrated management of adolescent and adult illness (IMAI) district clinical manual [14], WHO hand hygiene guidelines [15], WHO standard precautions [16]. Technical guidelines for integrated disease surveillance and response (IDSR) [17]). This process included iterative versions of the curriculum and a pilot in Lofa County. The result of this process was a locally relevant curriculum that could be used for both clinical and non-clinical workers that accounted for low literacy rates among non-clinical workers such as through an oral examination for this group. The national rollout was initiated with County health teams (CHTs) recommending county-level implementing partners; partners recruited were those already involved in EVD response in the country. This was followed by a central MoH-WHO training of trainers (ToT), county level ToTs and finally the frontline health workers’ training. In collaboration with the CHT, the lead implementing partner was responsible for county level training coordination and implementation, as well as managing other county based implementing partners. At the end of 2015, MOH in collaboration with WHO held a meeting for all implementing partners to discuss results, lessons learnt and recommendations.

**Overview of the rapid response team training and simulations:** NPHIL in collaboration with the epidemic preparedness and response (EPR) consortium (consisting of the international rescue committee, international organization on migration, international mercy corps, save the children) and WHO developed the county (sub-national) rapid response team (CRRT) training curricula and simulations in mid-2016 based on the integrated disease surveillance and response guidelines, the national epidemic and preparedness plan 2016, experiences and lessons learnt from the various outbreaks [18]. Modules included introduction to key concepts of IDSR, EPR and RRT, composition, roles and responsibilities of CRRTs. The two-day county simulations were designed to assess Ebola response capacity and gain outbreak experience in a safe environment. The exercise was undertaken at the health facility, isolation unit, district, and county levels: day one of the simulation was a functional exercise which assessed the capacity of the health facility staff, isolation unit staff, and the district health team (DHT). Day two of the simulation was a table top county integrated management system (IMS) exercise which evaluated the CHT response capacity. This was followed up in 2017 by the national RRT (NRRT) training package development, an amalgamation of the county RRT curriculum, and adaption of the WHO National RRT Training package [19]. On 29th May 2017, National level underwent RRT training that was divided into two parts: an orientation and two-day real-time simulation. The simulation involved mock patients presenting at various health facilities in Montserrado County, testing the entire response system: from health facility to county notification to activation and deactivation of national response team, and ended at the newly established Redemption Hospital 9-bedded integrated severely infectious treatment unit (InSitu); Liberia’s first official isolation facility.

Study design and data collection: survey data on confidence…

**Data analysis:** data sources include pre and post tests given to ETU and SQS training participants and programmatic database for the RRT training. Frequencies and percentages were used to describe data collected on the number of participants, both overall and at the county-level for the ETU and SQS training programs. For the ETU training, changes in confidence and knowledge between pre-and post-training tests were analyzed using paired t-tests. The analysis sample was limited to individuals who completed both pre- and post-tests. Results of the statistical tests were reported with the corresponding mean changes and 95% confidence intervals. A p-value < 0.05 was considered statistically significant.

**Ethical considerations**: confidentiality and anonymity of participants have been ensured.

## Results

Over a period of two and a half years (from September 2014 through to April 2017), there were 21,248 participants trained in the ETU, SQS and RRT trainings, with individuals participating in multiple programs.

### Healthcare workers in Ebola treatment unit

**Figure 2:**
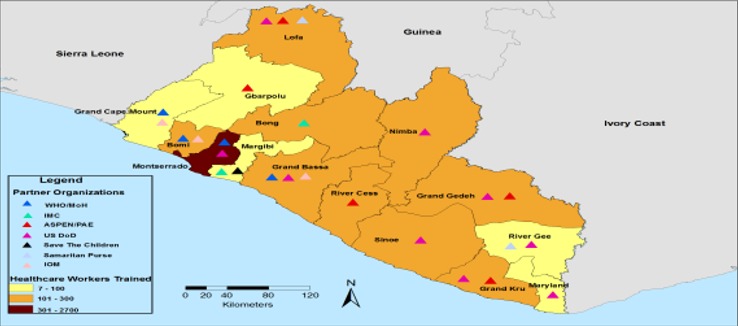
Healthcare worker Ebola treatment unit (ETU) training coverage by partners and counties

**Who was trained and where training occurred:** the Ebola healthcare worker training was conducted by seven organizations in Liberia-MOH in collaboration with WHO (WHO/MOH), International Organization for Migration (IOM), Medecin Sans Frontieres (MSF) Belgium, United States of America department of defense (US DoD), international medical corps (IMC), ASPEN medical and samaritan purse (Figure 2). For national HCWs, county coverage varied with the highest being from Montserrado (approximately 60 % of all HCWs trained), compared to other counties (which averaged 20-30%).

**Numbers trained:** from September 2014 to March 2015, a total of 5518 national and international health care workers were trained in the Healthcare Workers in Ebola treatment unit program; international HCWs contributed 16.7% (n = 923) and national HCWs contributed 83.3% (n = 4595) of total trained. The international cohort included more than 30 international partners or foreign medical teams (FMTs) including ECOWAS/African Union (AU), Bangladesh armed medial core, Cuban medical team, China armed forces, German red cross/armed forces, ARC, Heart to Heart International, IOM, International Rescue Committee (IRC), Save the Children, Partners In Health (PIH), US DOD, Swedish Civil Contingencies Agency, amongst others. The trainees consisted of slightly more non-clinicians (58%) than clinicians (42%). Trained participants supported not only clinical care delivery teams within ETUs, but also county training teams and critically re-opening and establishing IPC measures (especially linking to triage) in routine healthcare facilities. The best performing nationals were recruited and mentored as facilitators upon successful completion of the training to build national capacity for future outbreaks, trainings and refreshers.

**Figure 3:**
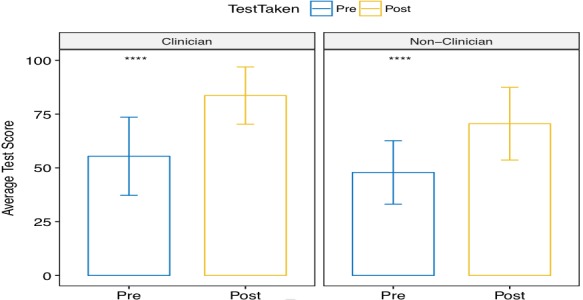
Average results of pre- and post-test assessments for ETU training participants

**Pre-and post-training knowledge assessment:** each cohort undertook a pre- and post-test assessment of their knowledge in the key taught areas. Pre-training scores were particularly low for PPE donning and doffing, EVD Clinical care and EVD laboratory diagnosis. Both clinicians (n = 188) and non-clinicians (n = 149) showed statistically significant improvements in knowledge on clinical care and IPC concepts, as measured by the 9-item pre-and post-test questionnaires (both p < 0.001). The average change in knowledge was significantly higher for clinicians than for non-clinicians (p = 0.006); specifically, the mean changes between pre- and post-test were 28.2 and 22.7-point changes for clinicians and non-clinicians, respectively (Figure 3).

**Figure 4:**
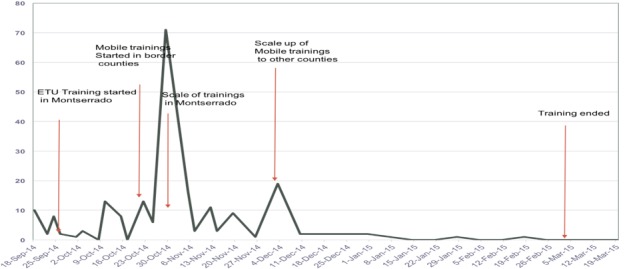
HCWs Ebola Virus Disease infection trend September 2014-March 2015

**Healthcare worker infections:** an associated finding that occurred during the training period was the downward trend in EVD healthcare worker infections between October 2014 and March 2015; healthcare worker infection rate was 9% by October 2014 and had dropped to 1% by January 2015 [1]. Furthermore, after the conclusion of training in March 2015, no healthcare worker infections were reported among those exposed to the confirmed cases despite the resurgence of Ebola cases in June and November 2015, and April 2016 (Figure 4).

### SQS Training

**Who was trained and where training occurred:** SQS was rolled out in all 15 counties by the respective CHTs in collaboration with the assigned implementing partners (19 in total; with a minimum of one and maximum of seven implementing partners per county).

**Numbers trained:** a total of 14,913 HCWs across all 15 counties were trained; 693 facilitators, 4099 clinicians, and 10,121 non-clinicians. The greatest number of trainees came from Montserrado, followed by Nimba and Bong, which are the three most populous counties. The ratio of trainees to facilitators varied across counties.

**Figure 5:**
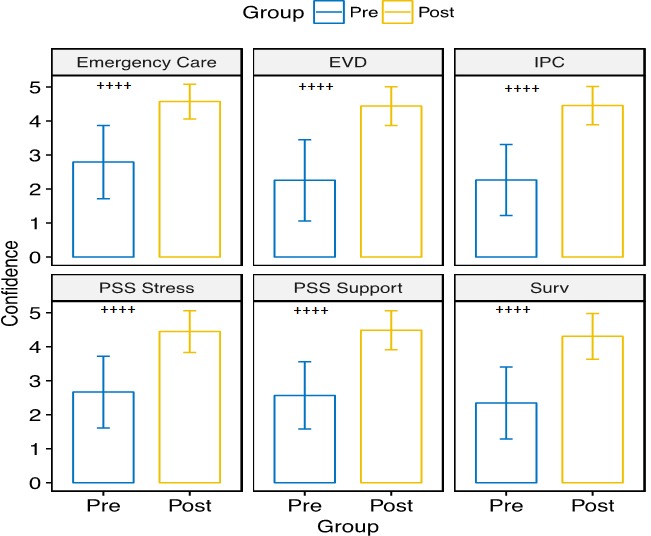
Pre- and post-test confidence scores for clinician subgroups who had the SQS training; bar height represents mean scores at pre- (blue) or post-test (yellow), given a 5-point Likert scale measuring confidence in various areas of the health sector; error bars reflect one standard deviation above and below the mean; mean confidence score in all areas significantly increased between pre- and post-test (++++p < 0.001)

**Figure 6:**
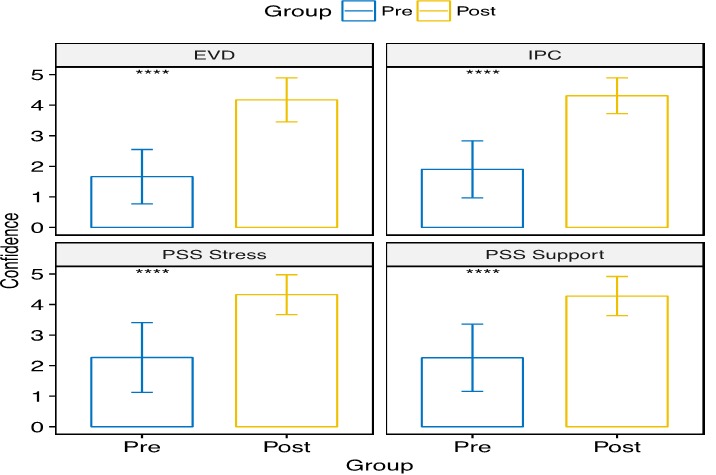
Pre-and post-test confidence scores for non-clinician subgroup who had the SQS training; bar height represents mean scores at pre- (blue) or post-test (yellow), given a 5-point Likert scale measuring confidence in various areas of the health sector; error bars reflect one standard deviation above and below the mean; mean confidence score in all areas significantly increased between pre- and post-test (++++p < 0.001) clinicians were tested on two additional indicators, relative to non-clinicans

**Pre-and post-training knowledge assessment:** each county-level cohort undertook a pre-and post-test assessment of their knowledge in the key subject areas. Pre-existing knowledge tended to be higher in the non-clinician cohort than in the clinician cohort. The knowledge gained among clinicians ranged from 15% to 48%; the highest gain was in the EVD, and surveillance modules and the lowest was in the IPC module. The knowledge gained among non-clinicians ranged from 14% to 23%; the highest gain was in the IPC module, and the lowest was in the EVD module. Participants ranked their confidence in key aspects of health system using a 1-5 Likert scale. Confidence in the areas of emergency medicine, EVD, IPC, psychosocial and surveillance increased significantly (all p < 0.001) between pre-test and post-test for both clinician and non-clinician groups (Figure 5 and Figure 6). Mean changes for confidence in emergency medicine and surveillance were 1.78 (95% CI: 1.60-1.96) and 1.96 (95% CI: 1.79-2.13), respectively, for clinicians. Mean changes for confidence in EVD, IPC, psychosocial support, and psychosocial stress were 2.18 (95% CI: 2.00-2.36), 2.19 (95% CI: 2.01-2.37), 1.92 (95% CI: 1.77-2.07), and 1.78 (95% CI: 1.60-1.96), respectively, for clinicians. For non-clinicians, mean changes for confidence in EVD, IPC, psychosocial support, and stress were 2.51 (95% CI: 2.40-2.63), 2.41 (95% CI: 2.30- 2.51), 2.02 (95% CI: 1.89-2.14), and 2.05 (95% CI: 1.93-2.18), respectively.

### RRT Training and Simulations

**Who was trained and where training occurred:** for the CRRT, the county and district health teams were trained in each of the 15 counties and 91 districts respectively in June and July 2016. National RRT training and simulation were held in Monrovia in May 2017.

**Numbers trained:** for the CRRT, 53 experienced outbreak personnel were initially trained as trainers, whom facilitated the county RRT training rollout for which a total of 792 individuals participated from all respective counties (93% coverage of targeted number). For the NRRT training there were 25 participants with representation from the various response pillars (epi-surveillance, case management, psychosocial, etc.)[20].

**Pre-and post-training knowledge assessment:** the results of the pre-tests administered to all participants in all 15 counties ranged from approximately 50-62%, while the post-test results ranged from 80-90%.

**Simulation findings: observations from the 15 county simulations undertaken included:** variable response capacity across counties (linked to previous EVD outbreak experience), the biggest gaps were at health facility level (inappropriate screening, isolation and case management) and district level (fragmented response), and county IMS was typically the strongest portion of the response, although pillars didn’t have a full understanding of roles and responsibilities. Key observation at the NRRT simulation was that participants were familiar with some but not many of the outbreak response processes. Key gaps identified included: information management via the different response levels, lack of familiarization of forms needed to be completed during an alert case, infection prevention and control measures (especially doffing), correct referral pathways, suspect case definitions and clinical management.

## Discussion

Pre-EVD, no formal EPR trainings were available in Liberia; as the EVD outbreak escalated in 2014 a dire need for HCWs capacity and confidence building was identified. To address this, a HCW training programme was initiated by the Ebola IMS using available resources for outbreak response and at the same time strengthening preparedness capacity. The implementation of these training programs led to heightened knowledge, skills and confidence amongst healthcare workers. There was a drop in HCWs infection following the introduction of the ETU training and in the post-EVD period no HCWs infections have been documented. In the post-EVD period, Liberia has experienced improvements in epidemic preparedness and response, as has been demonstrated by reduced response time to public health events: in 2016, out of 32 outbreaks, 44% (14/32) were responded to within 48 hours, whereas in 2017 there were 39 outbreaks of which 82% (32/39) were responded to within 48 hours [21]. The progress made by Liberia in epidemic preparedness and response can be attributed to capacity building in surveillance, laboratory diagnostics, case management, and workforce development including IPC that were achieved through training programs such as ETU, SQS and RRT. As a result of the trainings, guidelines have been developed and referenced during subsequent outbreaks and refresher training. This strategy corresponds with outbreak control measures implemented in experienced EVD countries such as Uganda [22]. The undertaking of the ETU and SQS training programs in the context of a widespread outbreak of a disease, highly transmissible in the event of direct contact and without well-established curative treatment, is noteworthy. The successful implementation was attributable to national and county MOH leadership and coordination, multi-stakeholder involvement with clear roles and responsibilities, a phased approach, iterative process of adapting training material based on implementation feedback, external quality assurance and resource availability.

A limitation of this study is that the reduction in HCWs infections following the ETU training cannot be attributed directly to the training only, due to concurrent in-country programs such as the influx of PPE supplies, which allowed for increased use independent of the training. Another weakness is the latct of data, particularly on RRT training, limited data analysis and what can be extrapolated from that specific training. These findings are consistent with the limited published literature [23]. this study validates that training programmes can be effectively developed and implemented during a crisis. Rapid implementation of large-scale training such as the SQS is possible with strong leadership, coordination and collaboration between the Ministry of Health (at all levels) and implementing partners. However, institutionalizing training in pre-service and academic institutions, and consistent in-service supportive supervision and simulations, and table-top exercises. In addition, for surge capacity it is critical to maintain an updated are key for sustainability and improve outbreak detection [3, 24].

## Conclusion

These training programmes, which were made possible thanks to EVD outbreak response resources, led to heightened knowledge, skills and confidence amongst healthcare workers, and more prompt response time to subsequent public health events. Training is critical for strengthening local capacity to contain outbreaks and prevent future ones, improve quality of care, and increase overall knowledge. Moreover, the timing of trainings and the use of refresher trainings should be part of an integrated programme to ensure up-to-date competencies, readiness and sustainability.

## What is known about this topic

- Healthcare workers training, including refresher training, during outbreaks is key to controlling disease transmission, protecting healthcare workers, patients and improving confidence in the healthcare system.

## What this study adds

- There is have been no healthcare workers’ infection in Liberia since completion of these training programs;
- Training programmes initiated during and post outbreaks can be leveraged as an entrance point to establishing an epidemic preparedness and response framework (including political buy-in and funding).

## Competing interests

The authors declare no competing interest.
